# Harmonizing Methods for Estimating the Impact of Contraceptive Use on Unintended Pregnancy, Abortion, and Maternal Health

**DOI:** 10.9745/GHSP-D-17-00121

**Published:** 2017-12-28

**Authors:** Ian Askew, Michelle Weinberger, Aisha Dasgupta, Jacqueline Darroch, Ellen Smith, John Stover, Melanie Yahner

**Affiliations:** a Population Council, New York, NY, USA. Now with the World Health Organization, Geneva, Switzerland.; b Marie Stopes International, Washington, DC, USA. Now with Avenir Health, Washington, DC, USA.; c Marie Stopes International, London, UK. Now with the United Nations Population Division, New York, NY, USA.; d Guttmacher Institute, New York, NY, USA.; e Palladium, Washington, DC, USA.; f Avenir Health, Washington, DC, USA.; g EngenderHealth, New York, NY, USA. Now with Save the Children, Fairfield, CT, USA.

## Abstract

Estimates of the potential impacts of contraceptive use on averting unintended pregnancies, total and unsafe abortions, maternal deaths, and newborn, infant, and child deaths provide evidence of the value of investments in family planning programs and thus are critically important for policy makers, donors, and advocates alike. Several research teams have independently developed mathematical models that estimate the number of adverse health outcomes averted due to contraceptive use. However, each modeling approach was designed for different purposes, and as such the methodological assumptions, data inputs, and mathematical algorithms initially used in each model differed; consequently, the models did not produce comparable estimates for the same outcome indicators. To address this, a series of expert group meetings took place in which 5 models—Adding it Up, Impact 2, ImpactNow, Reality Check, and FamPlan/Lives Saved Tool (LiST)—were reviewed and harmonized where possible. The group identified the main reasons for the inconsistencies in the estimates generated by the models for each of the adverse health outcome indicators. The group then worked together to align the methodologies for estimating numbers of unintended pregnancies, abortions, and maternal deaths averted due to contraceptive use, and reviewed the challenges with estimating the impact of contraceptive use on newborn, infant, and child deaths, including the lack of a conceptually clear pathway and rigorous evidence. The assumption that most influenced harmonization was the comparison pregnancy rate used by the models to estimate the counterfactual scenario—that is, if women who are currently using contraception were not using a method, how many would become pregnant? All the models now base this on the number of unintended pregnancies among women with unmet contraceptive need, bringing the estimates for unintended pregnancies, total and unsafe abortion, and maternal deaths much closer together. The agreed approaches have already been adopted by the Family Planning 2020 (FP2020) initiative and Track20, a project that supports FP2020. The experts will continue to update their models collaboratively to ensure that the most current estimation methodologies and data available are used. Valid and reliable methodologies for estimating these impacts from family planning are critically important, not only for advocacy to sustain resource allocation commitments but also to enable measurement and tracking of global development indicators. Conflicting estimates can be counterproductive to generating support for family planning programs, and this harmonization process has created a more unified voice for quantifying the benefits of family planning.

## Abstract (precis)

Five models estimate the impact of family planning on health outcomes, but the estimates previously have diverged because the models used different assumptions, inputs, and algorithms. After a collective harmonization process, the models now produce more similar estimates although they retain some minimal differences. These models assist in planning, resource allocation, and evaluation.

## BACKGROUND

The London Summit on Family Planning in 2012, and the ensuing Family Planning 2020 (FP2020) initiative,^1^ has mobilized substantial resources to support the expansion of family planning services. Specifically, the initiative aims to enable an additional 120 million women in 69 countries to use modern contraception by 2020 compared with the total number of users of modern contraceptive methods in 2012. The primary purpose for mobilizing these resources is to enable women to protect themselves against an unintended pregnancy, defined here as a pregnancy that was not wanted at all (i.e., unwanted) or that occurred earlier than intended (i.e., mistimed).

Reducing unintended pregnancies is frequently stated as a policy goal by governments, donors, and service delivery organizations. For example, the UK's *Framework for Results for Improving Reproductive, Maternal and Newborn Health in the Developing World* has a strategic priority to “prevent unintended pregnancies by enabling women and adolescent girls to choose whether, when and how many children they have” ^2^; one of the core development objectives of the United States Agency for International Development (USAID) is to “prevent 54 million unintended pregnancies” ^3^; and FP2020 tracks and reports annually on the estimated number of unintended pregnancies averted due to use of modern methods of contraception.^4^

Protection against an unintended pregnancy through use of contraception also potentially averts several adverse health outcomes that may occur had the pregnancy happened. These outcomes can include an unsafe abortion if the unintended pregnancy had been terminated; morbidity or death if the woman had suffered complications related to or aggravated by pregnancy; and morbidities or deaths of newborns, infants, and children if the pregnancy had resulted in a live birth. Clearly, it is not possible to *observe* and *measure* these outcomes directly because the unintended pregnancies have not occurred. However, being able to *estimate* these potential impacts is critically important for policy makers and donors because such estimates provide evidence of how family planning contributes to maternal and child health, thus providing strong advocacy messages to support investments in family planning from national and global funding sources. These estimates also demonstrate the link between use of family planning and achievement of the Sustainable Development Goals.^5^

Different estimates of the impact of contraceptive use on health outcomes has confused policy makers, managers, and donors, even when the reasons for the differences are explained.

The impact of reducing unwanted pregnancies on national fertility rates, and consequently on economic and social development including through a “demographic dividend,” has been well documented. In contrast, efforts to estimate the broader impact of contraceptive use on maternal, infant, and child health are less mature. But several models have been developed recently to estimate the impact of contraceptive use on averting adverse health outcomes. The purpose of this article is to describe these models, including how they are similar and different, and to report on collaborative efforts to better align the assumptions and inputs used in the models in order to produce more comparable results.

## CHALLENGES IN ESTIMATING THE HEALTH IMPACTS OF FAMILY PLANNING

**FIGURE 1.**
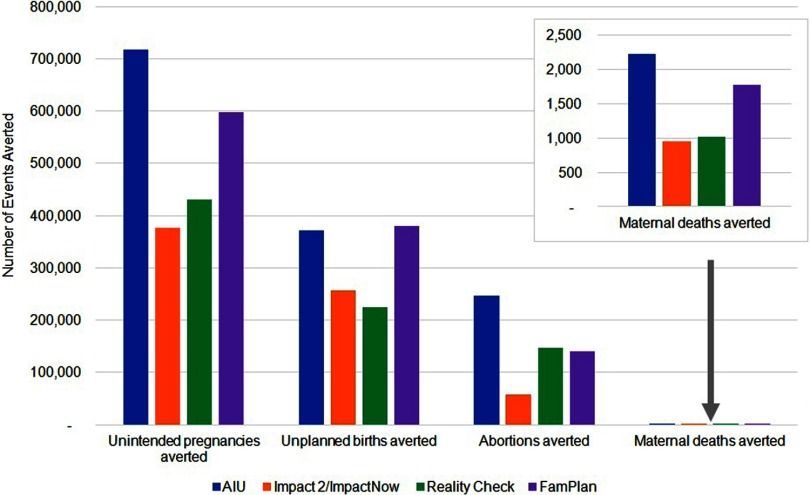
Different Estimates of the Health Impact of Contraceptive Use in Malawi From 5 Models, Before Harmonizing the Modeling Approaches

Abbreviation: AIU, Adding It Up.

Over the past few years, several research teams have independently developed models that estimate the number of adverse health outcomes averted due to contraceptive use. The development of these estimation models has been uncoordinated, however, with each approach being conceptualized and designed for complementary, yet different, purposes. Because the methodological assumptions for each model have differed, the data inputs required and mathematical algorithms used for each model have varied, and so the models did not produce comparable estimates for the same outcome indicators. For example, using the same dataset from Malawi, Figure 1 depicts estimates from 5 models for the numbers of unintended pregnancies, unplanned births, and abortions averted due to women using family planning. The generation of different estimates for the same indicator has confused policy makers, managers, and donors, even when the reasons for the differences are explained.

Each estimation model has been designed to serve a particular purpose and for different audiences, and so it would not be appropriate or desirable to try to consolidate them into 1 model. However, the scale of the differences shown in Figure 1 has raised concerns as to whether the assumptions for the models are sufficiently aligned. It has also highlighted the need to better communicate to decision makers the different purposes of each model so that they can select the appropriate model to generate the type of data needed to inform a particular decision.

To address these concerns, the Population Council's consortium for *Strengthening Evidence for Programming on Unintended Pregnancy* (STEP UP), funded by UKAID from the Department of International Development, convened a series of expert group meetings in September 2013, March 2014, and December 2014 to review and harmonize, where possible, the 5 most commonly used modeling approaches:

1. Adding It Up from the Guttmacher Institute
2. Impact 2 from Marie Stopes International
3. ImpactNow from the USAID-supported Health Policy Project
4. Reality Check from EngenderHealth's USAID-supported RESPOND Project
5. FamPlan and the Lives Saved Tool (LiST) from the Spectrum suite of models hosted by Avenir Health

Participants at these meetings included those responsible for designing and using these 5 approaches as well as recognized experts in demography, forecasting and modeling, and program evaluation. This was the first time that representatives from all 5 modeling teams had met together. The meetings provided a unique opportunity for the modelers to openly discuss the strengths and weaknesses of their modeling approaches among their peers in a neutral setting hosted and moderated by an organization that does not have a vested interest in any particular modeling approach.

A series of expert group meetings was convened to review and harmonize, where possible, the 5 most commonly used modeling approaches to estimate the impact of contraceptive use on health outcomes.

The initial meetings identified and highlighted the main reasons for the inconsistencies in the estimates generated by the models for each of the key adverse health outcome indicators (unintended pregnancy, abortion, maternal death, infant death, and child death). The group then worked together to seek and reach agreement on alignment of 4 methodologies:

1. Methodology for estimating the number of unintended pregnancies averted due to contraceptive use, including the comparison pregnancy rate to use for the counterfactual of contraceptive use (e.g., if women had not been using contraception, how many would have become pregnant)^6^ and failure rates to use for each type of contraceptive method (defined as the probability of a woman using a method becoming pregnant during 12 months of use^7^)
2. Methodology for estimating the number of abortions averted due to contraceptive use
3. Methodology for estimating the number of maternal deaths averted due to contraceptive use
4. Methodology for estimating the numbers of infant and child deaths averted due to contraceptive use

The expert group worked together to align the methodologies used in their models to estimate the number of unintended pregnancies, abortions, maternal deaths, and infant and child deaths averted due to contraceptive use.

This article will describe the purpose for each estimation model, review how each model used the 4 methodologies, and report on the alignments achieved through the collaborative process. (For a more detailed description and comparison of the 5 modeling approaches, see STEP UP, 2014.^8^) It is important to bear in mind that these are estimation models, which are used to generate measures for events that cannot be empirically observed and so they cannot be empirically validated either.

## PURPOSE OF EACH MODEL

The Guttmacher Institute's ***Adding It Up*** model uses tabulations from the most recent sources to estimate need, coverage, cost and impacts of modern contraceptive services, maternal and newborn health care, antiretroviral care for pregnant women living with HIV and their newborns, and treatment for 4 common sexually transmitted infections; varying scenarios of coverage and the costs and impacts across different levels of coverage are estimated. These scenarios and impacts are estimated individually and for combinations of these service needs. Impacts are expressed in terms of unintended pregnancies and their outcomes, including unplanned live and stillbirths, induced abortions and miscarriages, maternal and neonatal deaths and disability-adjusted life years (DALYs), and transmission of HIV to newborns. Adding It Up estimates have been calculated using multiple country-level datasets in Excel files, although most results are reported for geographical and other groupings of countries because of frequent limitations in the quality of original datasets. Although Adding It Up does not provide a template model, results and detailed tabulations are made available widely.^9^

The Guttmacher Institute's Adding It Up model estimates need for and cost of modern contraception, maternal and newborn health care, antiretroviral care for pregnant women living with HIV and their newborns, and treatment for 4 common sexually transmitted infections.

**Impact 2** is a spreadsheet-based model developed by Marie Stopes International that is designed to use existing service provision data. It can be used to estimate the impact of family planning, safe abortion, or postabortion care services provided by a particular organization or across an entire country. Impact 2 can estimate past, current, and future contributions of a service provision program to the additional number of contraceptive users and increases in contraceptive prevalence. It also estimates the wider health, demographic, and economic impacts of these services. In addition, Impact 2 can be used to estimate the quantity of service utilization needed to reach a goal, as well as to monitor progress over time. It has been used by managers of service delivery programs and for planning national strategies by governments.^10^

Marie Stopes International's Impact 2 model uses existing service provision data to estimate the health, demographic, and economic impacts of family planning and other services.

**ImpactNow** is a spreadsheet-based model developed by the USAID-supported Health Policy Project that estimates the health and economic impacts of family planning in the near term (2- to 7-year time horizon). It is designed to model the impacts of different policy scenarios and to compare the results of those scenarios in advocacy materials. It can help to estimate the impacts of many “what if” questions about policy options. The outcomes are focused on both reproductive health and economic metrics. Model results have been used to advocate for family planning programs in a number of countries, primarily in sub-Saharan Africa.^11^

The Health Policy Project's ImpactNow model estimates the health and economic impacts of family planning in the near term.

Designed by EngenderHealth under the USAID-funded RESPOND Project, **Reality Check** is a Windows application for use in low-resource settings that can be used to set family planning goals and plan for service expansion to meet those goals; it can also provide advocacy data by estimating program requirements for implementation, along with the impact of achieving contraceptive goals. The tool enables users to test future goal scenarios, including changes in the method mix, and to compare those future scenarios with past performance to determine whether current goals are realistic. Reality Check can be used at any geographic level for which population and contraceptive prevalence rate data can be defined. The tool has been used to establish evidence-based family planning goals and to inform holistic plans to meet those goals in several sub-Saharan African countries.^12^

EngenderHealth's Reality Check can be used to set family planning goals and plan for service expansion to meet those goals.

**FamPlan** and **LiST** are modules in the Spectrum modeling system. FamPlan^13^ estimates the family planning requirements to meet goals, such as reducing unmet need, and the consequences of scaling up contraceptive use, in terms of outcomes such as fertility, births, and unintended pregnancies. The module uses the proximate determinants of fertility to model the impact of change on the total fertility rate (TFR), and then makes demographic projections of the resulting population size and structure. At the time this work was taking place, FamPlan did not estimate impacts averted but rather events that happen (e.g., births, abortions); however, by comparing multiple projections one could arrive at estimates of impacts averted. (FamPlan has now been updated with an option to calculate the number of unintended pregnancies, total and unsafe abortions, and maternal deaths averted.)

FamPlan, from the Spectrum modeling system, estimates family planning requirements to meet specific goals and the consequences of scaling up contraceptive use in terms of health outcomes.

LiST^14^ supports the development of plans for child survival programming by estimating the current distribution of child deaths by cause and the effects of health interventions, including family planning, on child mortality rates. Both the LiST and FamPlan modules have been applied by a large number of countries to develop national family planning and child survival plans and for global analyses for planning and resource mobilization.

LiST, also from the Spectrum modeling system, estimates the current distribution of child deaths by cause and the effects of health interventions on child mortality rates.

## METHODS FOR ESTIMATING HEALTH OUTCOMES

### Estimating Unintended Pregnancies Averted

In all of the models except FamPlan, a pregnancy rate is used to estimate the counterfactual scenario—that is, if women who are currently using contraception were not using a method, how many would become pregnant? Including this is important because not all contraceptive users will become pregnant if they are not using contraception. The FamPlan model generates and compares multiple scenarios, rather than directly estimating numbers of pregnancies, by using the proximate determinants model to estimate the impact of contraceptive prevalence rate changes on the TFR. Following this harmonization process, a new option has been added to FamPlan to allow for estimation of impacts using the counterfactual approach employed by the other models.

Moreover, the expert group reached agreement on the particular pregnancy rate variable that should be used as a default in all the models; prior to this harmonization process, the models had been using different pregnancy rates (see below). The default value in all models is now a “non-user at risk of unintended pregnancy,” which serves as a proxy for the counterfactual. The group defined this unintended pregnancy rate as the likelihood of a pregnancy over 12 months for sexually active, fecund women who do not want to become pregnant and are not using contraception–that is, among women with an unmet need for contraception. The models use the Demographic and Health Survey (DHS) definition of unmet need because it is widely understood and facilitates comparisons across datasets, time periods, and countries. This definition assumes sexual activity among married women.

The group agreed to use the pregnancy rate estimated using the Adding It Up methodology,^9^ which divides the number of unintended pregnancies among women with unmet need (i.e., non-users at risk of unintended pregnancy) by the total number of women with unmet need. This methodology estimates the pregnancy rate among women who do not wish to become pregnant, whereas the previous comparison pregnancy rates used by ImpactNow, Impact 2, and Reality Check had been for all women not using contraception. The agreed-upon pregnancy use estimates are based on (1) country-level data for the numbers of women by contraceptive need and use; (2) typical-use failure rates among developing country and U.S.-based contraceptive users; and (3) Adding It Up's sub-regional estimates of numbers of unintended pregnancies. The global pregnancy rate is the median of these country-specific pregnancy rates (which at the time of the meeting was 31%, with an interquartile plausibility range of 23% to 38%). Previously, the various models had used pregnancy rates of either 85%, representing the pregnancy rate among women in the United States who stopped using contraception to become pregnant,^15^ or 40%, representing a previous revision to the 85% rate to capture a rate of women not actively trying to get pregnant. Thus, moving to the agreed rate has led to substantial changes in the results from some models. Furthermore, the group also agreed to use one global pregnancy rate rather than trying to estimate potential regional or country variations.

Because no method is 100% effective at preventing pregnancy and because methods may not always be used correctly and consistently, “typical-use method failure rates,” are included in the models. The typical-use failure rate is the probability of pregnancy during a specified time period among women using a method as *typically* used (i.e., not necessarily correctly and consistently, which is referred to as the “perfect-use” failure rate).^16^ The original versions of all models used several sources for method-specific typical-use failure rates, largely based on research by Cleland, Ali, and Shah^17^ (using data from DHS, which includes non-permanent method users only) and by Trussell^15^ (for data on permanent method users from clinical trials with large study populations). The group had concerns about mixing failure rates estimated differently and for different populations but agreed to continue to use both sources. It was acknowledged that more needs to be known about method effectiveness, consistency, and correctness of use, and how these affect method effectiveness. The group committed to consider further evidence as it emerges and to coordinate updating assumptions and data inputs to continue improving the models. For example, recent estimates of failure rates in 43 developing countries using DHS data were published in 2016^18^ and could be reviewed for potential use in the models.

The group discussed whether method failure rates should be adjusted for individual countries. Failure rates by method are only available from a limited set of national datasets and as the only data available, these failure rates are generally accepted as being valid globally. Within the group, the Impact 2, ImpactNow, Reality Check, and FamPlan modelers decided to continue to use these “global” failure rates for all countries, whereas the Adding It Up approach will continue to use method failure rates that are adjusted against sub-regional estimates of unintended pregnancies.

### Estimating Abortions Averted

Using contraception will reduce the number of unintended pregnancies, which in turn will reduce the number of induced abortions—safe or unsafe—that some women use to terminate an unintended pregnancy. Previously, the models used different methodologies to estimate the number of abortions averted, leading to some very large discrepancies. Given the very limited data available on abortion, it is difficult to have a clear methodological approach for modeling this outcome. Recognizing the importance of harmonization, the group agreed that all 5 models would base their estimates on the sub-regional proportion of unintended pregnancies that end in induced abortion, using the rates published by Sedgh et al. in 2014.^19^ The number of abortions averted by use of modern contraception is thus estimated as the number of unintended pregnancies averted by use of modern contraception multiplied by the proportion of unintended pregnancies that end in induced abortion; the number of induced abortions is estimated from available data, special country studies, and consultations with experts, including the World Health Organization (WHO). Recent estimates of abortion levels and safety can be used for future inclusion in the models.^20^^,^^21^

All 5 models now base their abortions averted estimates on the sub-regional proportion of unintended pregnancies that end in induced abortion.

Some unintended pregnancies end through spontaneous abortion (i.e., a miscarriage), and a very small proportion end in an ectopic pregnancy. Reducing the number of unintended pregnancies, therefore, will also reduce the number of miscarriages and ectopic pregnancies. Reliable statistics for these rates do not exist, however, and the models estimate these numbers slightly differently, but the differences are very minor. Therefore, no change was deemed necessary.

### Estimating Maternal Deaths Averted

The causes of maternal mortality are several. WHO defines a maternal death as^22^:

The death of a woman while pregnant, or within 42 days of termination of pregnancy, irrespective of the duration and the site of the pregnancy, from any cause related to or aggravated by the pregnancy or its management (from direct or indirect obstetric death), but not from accidental or incidental causes.

For the purpose of these models, a maternal death can be categorized according to whether the woman died from *complications of an induced abortion* or due to other *obstetric causes* during pregnancy, delivery, or up to 42 days after delivery.^23^

Estimates are available for the maternal mortality ratio (MMR) in each country—the number of maternal deaths during a given time period per 100,000 live births during the same time period.^24^ Applying the national MMR to the number of births averted would not be correct, however, because the national MMR reflects the risk of dying related to the national distribution of all pregnancies and their outcomes (live births, miscarriages, abortions). All of the models agreed to use the “percentage of unintended pregnancies ending in induced abortion” to estimate the number of abortions averted through use of family planning. As the distribution of outcomes of *unintended* pregnancies is different from that of *all* pregnancies, the MMR does not represent the risk of dying associated with an unintended pregnancy. Rather, a new MMR must be constructed to represent the risk of dying associated with an unintended pregnancy to account for the risk associated with each pregnancy outcome: live birth, miscarriage, and abortion.

The models have to construct a new maternal mortality ratio, different than the UN published estimates, that represents the risk of dying associated specifically with an *unintended* pregnancy.

The group recommended, therefore, that all of the models should estimate an unintended pregnancy MMR that is appropriate for each model. An estimation approach has been developed and is now being used by Impact 2, ImpactNow, and FamPlan. Further work is needed to understand the proportion of deaths due to unsafe abortion represented within the all-cause estimations by WHO,^25^ since those causes due to unsafe abortion are not always clearly recorded (e.g., a hemorrhage could be a result of unsafe abortion or non-abortion causes). The group agreed to work with WHO to better interpret these data and to consider further changes to this methodology, given the large differences in MMRs that remained in the models after the harmonization process.

### Newborn, Infant and Child Deaths Averted

For more than 3 decades, contraceptive use has been promoted as a key strategy for reducing newborn, infant, and child deaths. This causal relationship is most frequently conceptualized in terms of *risk reduction*: women using contraception can determine the timing and number of children so that they can reduce the likelihood of experiencing one of the 4 “toos”—having a birth when they are *too young*; having births spaced *too soon*; having *too many* births; and having a birth when they are *too old*. Evidence indicating that such births are generally *correlated* with adverse outcomes is well established through cross-sectional surveys,^26^^–^^33^ particularly for “too young” and “too soon” births and their correlation with newborn or infant death and stunted growth and development in children surviving such births.

To guide policy and programming advocacy and implementation, however, it is critically important that claims to *causal pathways* between contraceptive use, timing of births, and newborn, infant, and child mortality and morbidity be more fully understood and justified empirically. The estimates of newborn, infant, and, especially, child deaths averted due to contraceptive use are recognized as the weakest elements of all of these models. While the relationships between risky pregnancies (as defined above in the 4 “toos”) and newborn and infant mortality and morbidities, and with poorer child development, are relatively well supported with the evidence available, the correlation with *child mortality* is poorly understood because many other factors may intervene by the time of childhood (i.e., between 1 and 5 years old). The estimation models must produce valid measures based on logically consistent causal pathways in order for policy statements and initiatives advocating investments in family planning to legitimately cite quantified reductions in newborn and infant, as well as child, mortality as among the anticipated returns on investment for family planning. (FP2020 has chosen not to include these impact indicators in its analysis due to the uncertainties of attribution.) Consequently, there is some urgency to better understand the nature of these relationships to prevent any inappropriate expectations of causality.

The estimates of newborn, infant, and child deaths averted due to contraceptive use are recognized as the weakest elements of these models due to a lack of conceptually clear casual pathway and lack of rigorous evidence.

These pathways are being analyzed further using recent datasets and new analytical techniques to seek explanations for *biological mechanisms* that link high-risk births (the 4 “toos”) to adverse birth outcomes for newborns (e.g., small for gestational age, preterm births) and to infant mortality.^34^ They are also being analyzed for the *behavioral pathways* that include, for example, lower utilization of health services by women with many births, who have experienced unintended pregnancies, and with inequitable access.^35^^,^^36^

The lack of conceptually clear pathways based on rigorous evidence made it challenging for this group of modelers to agree on an approach for estimating such impacts. All models, except Reality Check, currently estimate these impacts; given the lack of agreement on conceptual pathways, the group agreed that each team would continue to use their own estimation methods. The group also agreed that descriptions of the models should make it clear if the impacts being modeled are based on reductions in risk, as is the case for Impact 2 and ImpactNow, or on the demographic impact of fewer pregnancies resulting in fewer deaths and morbidities, as is the case for Adding it Up. These different approaches are not comparable and produce estimates of differing orders of magnitude.

## RESULTS OF HARMONIZATION

**FIGURE 2.**
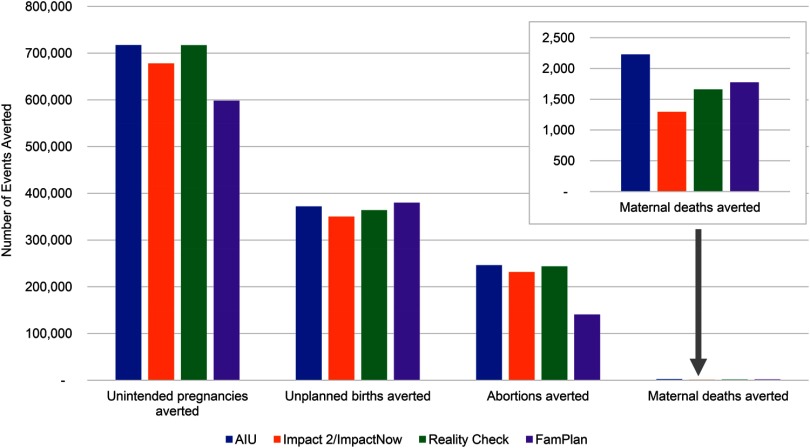
More Uniform Estimates of the Health Impact of Contraceptive Use in Malawi From 5 Models, After Harmonizing the Modeling Approaches

Abbreviation: AIU, Adding It Up.

Following the consultations, the models were re-run using the harmonized assumptions. As Figure 2 shows, the measures generated for the Malawi dataset are now much closer, indicating that the harmonization process was successful. It should be noted that in this example all models used the same input assumptions to assess whether, given the same assumptions, results would be comparable, thus isolating any differences due to methodologies and default data. It may not always be the case that all models are using the same input data as defaults. In addition, because methodological differences exist due to the different uses of the models, it is not possible to reach perfect agreement. However, comparing Figure 2 with Figure 1 shows that results have become much more consistent across all 4 estimated impacts.

After the harmonization process, the measures generated by the models are now much closer.

The assumption that most influenced harmonization was the comparison pregnancy rate used by the models. The group had agreed to use the comparison pregnancy rate being used by Adding it Up, which is why the Adding It Up outputs did not change considerably pre- and post-harmonization. Conversely, Impact 2/ImpactNow and Reality Check outputs changed the most because these models changed the comparison pregnancy rate that they had been using. FamPlan does not include this concept in its modeling calculations, but its outputs did change somewhat because it adjusted the contraceptive failure rates used in the model.

The assumption that most influenced harmonization was the comparison pregnancy rate used by the models.

## LIMITATIONS AND FURTHER IMPROVEMENTS

This consultative process provided an important and unique opportunity for those involved in the development of methodologies to estimate the health impacts of contraceptive use to convene and discuss the various approaches used. The group also discussed variations in the quality of the existing data and identified ways in which each model could take this variability into account. The process enabled the group to clarify and differentiate the intended use(s) for each model, to identify where and why similarities and differences exist, and to better align key assumptions for the modeling approaches where needed, while taking into account the critical differences in purpose and approach for each model. As a result of this process, consensus has been reached to ensure that, wherever possible, the models do not generate conflicting estimates that may confuse decision makers. A key lesson learned is the importance of making sure that decision makers understand the purpose of each model and select the model most appropriate for their needs. The group also agreed that each model was sufficiently different to warrant continued use of all models and not to attempt to combine or eliminate any of them.

It is important for decision makers to understand the purpose of each model and to select the model most appropriate for their needs.

The results reported in this article comparing the model outputs pre- and post-harmonization were generated in mid-2014; further work and updates have been done on the models since then. The experts will continue to update their models collaboratively to ensure that the most current estimation methodologies and data available are used and that any changes are made harmoniously. This alignment and consensus-building process has strengthened the models by enabling their developers to benefit from each other's experience and research. Moreover, decision makers and managers using the different models can more clearly understand the assumptions behind each model in order to make informed choices between them. This alignment process has shown that through transparent and participatory engagement it is possible to make concrete steps toward harmonization.

Further, the consensus approaches have been adopted by FP2020 to estimate 3 of its 17 core indicators: (1) unintended pregnancies averted by modern contraceptive use, (2) maternal deaths averted by modern contraceptive use, and (3) unsafe abortions averted due to modern contraceptive use. Moreover, Track20, a project of Avenir Health that supports FP2020, has developed a simple Excel-based tool to estimate these indicators following the agreements made during this harmonization process.

This process has also highlighted the need for continued research to better understand the causal pathways and to estimate the input parameters for each model, as well as the benefit of sustained cooperation within the modeling community to ensure that further methodological developments are shared to benefit all approaches. Four priorities identified by the group include:

- Further research to elucidate the relationships between contraceptive use, birth timing, and newborn, infant, and child mortality and morbidities
- Definition and measurement of pregnancy intendedness and whether and how intentionality impacts pregnancy and birth outcomes
- Definition and measurement of unsafe abortion-related maternal mortality, including identifying abortion-related mortality that is possibly subscribed to other causes (e.g., hemorrhage) and accounting for the impact of wider access to drugs such as misoprostol
- Consensus on method failure rates and the factors influencing variability across various sub-populations

## CONCLUSION

Contraception enables women and couples to achieve their fertility intentions by having the number of children they want, at the time they want. In addition to benefiting women individually and their families and communities, family planning also impacts a number of health outcomes, which substantially increases the return on investment in family planning programs. Consequently, valid and reliable methodologies for estimating these broader impacts are critically important, not only for advocacy to sustain family planning allocation commitments but also to enable measurement and tracking of global indicators for elements of the Sustainable Development Goals, and for strategic planning to reduce maternal, infant, and child mortality and morbidities. Conflicting estimates can be counterproductive to generating support for family planning programs, and this harmonization process has created a more unified voice for quantifying the benefits of family planning.

## Notes

**Peer Reviewed**

**Funding**: UKAid from the Department of International Development.
